# Moderating role of financial legal satisfaction in the relationship between business environment and health outcomes among Chinese financial professionals: a cross-sectional study

**DOI:** 10.3389/fpubh.2025.1705998

**Published:** 2026-01-16

**Authors:** Songze Lv, Xin Zhou, Yan Wang

**Affiliations:** 1 School of Law, Heilongjiang University, Harbin, China; 2 School of Foreign Languages and Literature (School of Region and Country Studies), Heilongjiang University, Harbin, China; 3 Department of Health Management, School of Health Management, Harbin Medical University, Harbin, China

**Keywords:** business environment, financial legal satisfaction, financial professionals, health outcomes, moderating role

## Abstract

**Background:**

As a forward-looking dimension of this study, the business environment presents novel avenues for optimizing the health outcomes of financial professionals. The study examines the relationships among business environment, financial legal satisfaction, health outcomes, and their moderating mechanisms.

**Methods:**

The study employed a cross-sectional design, utilizing a multi-stage stratified sampling approach to collect 514 valid responses from Chinese financial professionals (effective response rate: 71.49%). Hierarchical regression analyzed determinants of health outcomes and the moderating effects of legal satisfaction.

**Results:**

Chinese financial professionals exhibited the following health outcomes (*M* ± SD): sleep quality (3.33 ± 1.06), subjective health (3.50 ± 0.93), and psychological stress (2.50 ± 1.01). Financial legal satisfaction significantly moderated the associations, weakening the positive link between the business environment and subjective health (*β* = −0.625, *p* < 0.01). It amplified the negative association with psychological stress (*β* = −0.572, *p* < 0.05).

**Conclusion:**

Chinese financial professionals exhibit compromised health profiles, characterized by diminished sleep quality, impaired subjective health, and heightened psychological stress. Financial legal satisfaction plays a critical moderating role, underscoring the importance of aligning legal framework modernization with business environment reforms. Targeted legal optimization can systematically enhance workforce health by strengthening the infrastructure that supports the health outcomes of financial professionals.

## 1 Introduction

### 1.1 Health outcomes of financial professionals

Bolstered by World Bank support and driven by continuous innovation and reform of its domestic business environment within the business and finance sectors, China has made remarkable strides. Financial professionals, as an integral part of this economic landscape, have played a pivotal role in this development. Ning et al. (1) further underscores their critical importance, highlighting their influence on China’s economic trajectory. The global economic development and the deregulation of the labor market have jointly driven profound changes in the working and sales models of financial services, fostering new technologies and operational structures, which have harmed the health of financial professionals (2). Additionally, Marazziti et al. (3) indicates that prolonged global economic crises trigger severe recessions across major economies, intensifying financial sector competition, driving institutional reforms, advancing economic planning, and lowering inflation rates. Consequently, financial professionals confront mounting challenges, including unemployment, economic strain, heightened work intensity, and strained family relationships. As key players in risk prevention and control, financial professionals need to collaborate with regulatory authorities and relevant government departments to perform their duties, jointly bearing the responsibility of maintaining financial stability and promoting healthy economic and social development (4). Safeguarding their health outcomes and well-being is essential for national economic strategy, as healthy financial professionals may enhance financial technology innovation, organizational resilience, and financial market stability. Within the burgeoning realm of financial technology, prioritizing health outcomes may be a critical determinant for achieving social cost efficiency and amplifying the positive impact of the digital economy.

Health outcomes encompass multiple dimensions, and this study adopts three dimensions commonly utilized in public health (5), namely sleep quality, subjective health, and psychological stress. The health outcomes of financial professionals in China have raised significant concerns. In accordance with a statistically robust White Paper published by authoritative medical associations, 49.7% of financial professionals work 46–55 h weekly, 39.2% work 55–65 h, and 11.1% exceed 65 h (Chinese Medical Doctor Association & Chinese Hospital Association). A cross-sectional study (6) conducted in Liaoning Province, China, revealed that surveyed financial professionals exhibited a higher prevalence of depressive symptoms and generally poor mental health. The health outcomes of financial professionals have emerged as a pressing global public health concern. A cross-sectional study (7) of 2,500 employees at a national bank in Brazil indicated that those exposed to high work pressure are more susceptible to symptoms of depression and anxiety, with a comparatively higher detection rate of multiple personality disorder. The study by Mendola and Leoni et al. (8) examined the occupational health outcomes among Italian financial professionals following COVID-19-related work adjustments and reported a 17% increase in the use of psychotropic medications. Therefore, paying attention to their health outcomes is not only related to individual career risk prevention but also has positive significance for public health and occupational well-being, serving as an important support for maintaining the stable operation of the financial system. Against the backdrop of the current rapidly evolving business environment, coupled with market expansion, technology-driven innovation, and inherent characteristics of the financial industry, such as intense competition, significant challenges, and stringent working conditions, financial professionals are confronted with substantial pressure and numerous obstacles (9). Compounding these issues, the pervasive use of work-related information and communication technologies after hours in China (10) contributes to acute sleep disruption and emotional exhaustion. Moreover, a sedentary lifestyle, which is highly prevalent among office workers, is associated with various health impairments (11) that may substantially diminish financial professionals’ subjective perception of their health status. Given that financial professionals play a pivotal role in driving China’s financial reform and global economic prosperity, their health status warrants prompt attention, as addressing these health-related challenges is crucial for sustaining national economic development and international financial cooperation.

### 1.2 Internal relationships between business environment and health outcomes

The “Doing Business” concept emanates from the World Bank Group’s International Finance Corporation (IFC) survey, which was initiated in 2002 to assess the regulatory environments of small and medium-sized enterprises across economies. Through longitudinal comparisons of business regulations, it produces the annual Doing Business Report (DB Report), first published in 2003 (12). In contrast, the business environment constitutes a broader analytical framework that encompasses institutional factors across political, economic, social, and cultural dimensions (13). Compared with the former, it may have more theoretical depth and practical explanatory power. The term “business environment” received formal recognition in central government policies during the period from 2011 to 2017 (14). In 2020, China rose to 31st position in the World Bank’s Ease of Doing Business Index, reflecting significant improvements in its business environment. A high-quality business environment is the cornerstone of economic development. Recognizing this, the Chinese government has prioritized optimizing the business environment as a core component of comprehensive innovation and sustainable economic growth (15). Concurrently, China has established comprehensive framework standards for business environment, emphasizing the construction and improvement of an evaluation framework centered on the satisfaction of market entities and the public, and enhancing its guiding role in economic development (16). Additionally, the cultivation of new quality productive forces has emerged as a crucial growth driver, representing both a necessity for China’s high-quality development and a strategic approach to global competitiveness. An optimized business environment facilitates innovation and entrepreneurship, thereby accelerating the development of these advanced productive forces (17). Founded in 1952, the China Council for the Promotion of International Trade (CCPIT) functions as a national-level institution dedicated to promoting foreign trade and investment. The data disseminated by CCPIT bears substantial statistical significance. The China Business Environment Research Report (2024), with approximately 90% of surveyed enterprises expressing satisfaction (“highly satisfied” or “reasonably satisfied”), reflects a 2.1 percentage point increase from 2023 levels. Zheng et al.’s study (18) revealed an intricate and robust connection between the business environment and the financial industry. In the era of burgeoning digital transformation and innovation, digital finance has emerged as a linchpin, attracting significant global attention from the financial industry. Virglerová et al. (19) identify financial markets and banks as one of four key determinants of business environment quality. Capitalizing on state-of-the-art information technology, it delivers highly efficient, convenient, and secure financial services to individuals and enterprises (20). This evolving trend mandates the optimization of the urban business environment, thereby facilitating enhanced urban innovation and governance. The dynamic transformation of the financial industry’s business environment has indisputably exerted substantial impacts on financial professionals. They are subjected to considerable pressure stemming from the necessity for continuous skill enhancement, while also facing occupational uncertainties inherent in the digital finance era, alongside challenges arising from the growing demand for interdisciplinary expertise within the industry (21). Collectively, these elements are highly prone to detrimentally affect their health outcomes.

Triadic reciprocal determinism (TRD) provides a theoretical framework for examining the reciprocal relationships among individual behavior, personal determinants, and environmental factors. The theory elucidates the mechanism by which individuals dynamically adapt to changes in their environment in a systematic manner to achieve the desired outcome. Specifically, TRD demonstrates the dynamic interplay whereby individuals modify their behavior and environment while simultaneously being shaped by these same factors in pursuit of desired results and the avoidance of negative consequences (22). Guided by the triadic reciprocal determinism, the study will thoroughly analyze the complex mechanism by which the business environment impacts the health outcomes of financial professionals. With the acceleration of digital transformation, continuous innovation in financial service models and business scopes, and frequent changes in regulatory policies, the business environment is likely to undergo significant shifts, leading to a surge in demand for interdisciplinary talents in the industry. To adapt, financial professionals must continuously acquire new knowledge – including financial technology innovations and regulatory updates – while adjusting to novel business models, creating significant adaptive pressures. This behavior alleviates the pressure of adapting to a new environment; additionally, it may potentially exert a positive impact on the business landscape through skill enhancement. Nevertheless, owing to inexperience, financial professionals may adopt passive coping measures, thereby inducing anxiety regarding changes in regulatory policies, which may exacerbate physical and mental fatigue and ultimately deteriorate overall health outcomes. These include the cognitive load associated with absorbing new knowledge and the time–pressure-induced sense of urgency to adapt to the new business rhythm, which may all be potential risk factors for health outcomes.

Personal factors play a pivotal role in the relationship that exists between business environment and health outcomes. Financial professionals’ own working experience, education level, knowledge reserve, and other characteristics may affect their perception of business environment pressures and their response behavior. Seasoned financial professionals may take a positive attitude toward challenges and take the initiative to adjust the pace of work to relieve stress effectively. With a solid professional foundation, individuals with stronger academic backgrounds may exhibit better adaptation to environmental changes, experiencing reduced stress (23). Moreover, the dynamic evolution of the business environment is likely to exert a holistic influence on the health outcomes of financial professionals. It does so by propelling their behavioral factors, which are mediated by personal factors, thereby manifesting the “environmental factors - personal factors - behavioral factors” interactive pathway posited by the triadic reciprocal determinism theory. China’s ongoing reform and opening-up policies, coupled with the steady progress of the Belt and Road Initiative, continue to transform this environment. While these changes drive industry advancement, they concomitantly pose potential health risks to financial professionals. Nevertheless, the trajectory of optimizing and upgrading the business environment is inexorable. Investigating the underlying mechanisms for improving financial professionals’ health is therefore of substantial practical importance, particularly for maintaining talent stability and ensuring the industry’s sustainable development.

### 1.3 The moderating effect of financial legal satisfaction

Law, a system of social norms instituted by authoritative institutions and vested with coercive force, demonstrates divergent legal ramifications in various countries, owing to idiosyncratic factors such as cultural and political aspects. In China, under the influence of Confucian culture (24) and distinct political features, law serves as a robust governance instrument. The Communist Party of China (CPC) conceives of law in a manner distinct from liberal perspectives: it is neither positivist norms autonomous from politics nor unwritten constitutional principles, but rather the codification of the collective will of both the Party and the people, while simultaneously functioning as an institutional constraint mechanism (25). Law assumes a fundamental role in maintaining financial stability, optimizing economic efficiency, and safeguarding systemic integrity (26). The rapid development of fintech necessitates that legal systems continually adapt to address emerging challenges. Meanwhile, China is actively adjusting relevant regulations to align them more closely with the demands of financial innovation. For instance, Yu et al.’s study (27) indicates that China’s data protection statutes impose constraints on the functioning of the Financial Credit Information System (FCIS) to achieve a high level of protection, given that data transmissions to the FCIS hinge on the consent of the data subject. However, Yang’s study (28) identifies persistent challenges in China’s financial legal system, including legislative loopholes, rampant regulatory arbitrage, and erosion of financial consumer rights. Comprehensive financial legislative reform and institutional innovation are crucial to addressing these deficiencies and enhancing safeguards for both financial consumers and professionals.

A comprehensive assessment of the financial industry’s business environment should incorporate administrative, financial, and legal dimensions, particularly in emerging markets such as China (29). Empirical evidence demonstrates that, among business environment sub-dimensions, infrastructure contributes most significantly, followed closely by rule-of-law conditions (30). The study by Chen and Yang, which encompassed 3,088 manufacturing firms across 240 cities in China, further revealed that robust legal environments facilitate enterprise digital transformation, enhancing competitiveness and regional economic development (31). Recognizing the unique role of the legal environment in business environment, countries worldwide increasingly emphasize rule-of-law-based optimization. However, China’s approach differs substantially from Western models in terms of national culture, government role positioning, and economic system. Specifically, China has established a centralized political structure in which the government actively engages in market regulation, in contrast to the typically passive “night watchman” role of Western governments. At the level of economic system, China implements a socialist market economic system (32). Consequently, China has proactively established a rule-of-law-based business environment, recognizing legal governance as the fundamental determinant of business ecosystem quality, consistent with Bota’s identification of the rule of law as the most decisive factor in a business environment (33). Since 2012, China has focused on the bottlenecks in the construction of its own business environment, continuously improved relevant laws and regulations, intensively introduced a large number of pragmatic and effective policies and measures to continue to promote the construction of the business environment, actively promoted the implementation of high-level investment liberalization and facilitation policies, improved the level of opening up, increased services for enterprises, optimized the environment for foreign investment, and continuously enhanced the attractiveness of foreign investment. The Chinese government has explicitly committed to fostering a first-class business environment and achieving marketization, legalization, and internationalization (30), a commitment that is concretely implemented through regulatory frameworks. It is worth noting that “Regulations on Optimizing the Business Environment,” which was officially released in January 2020, is a landmark initiative. This policy represents the institutionalization of China’s accumulated experience in business environment reform, marking a significant milestone in its policy evolution (34).

**Figure 1:**
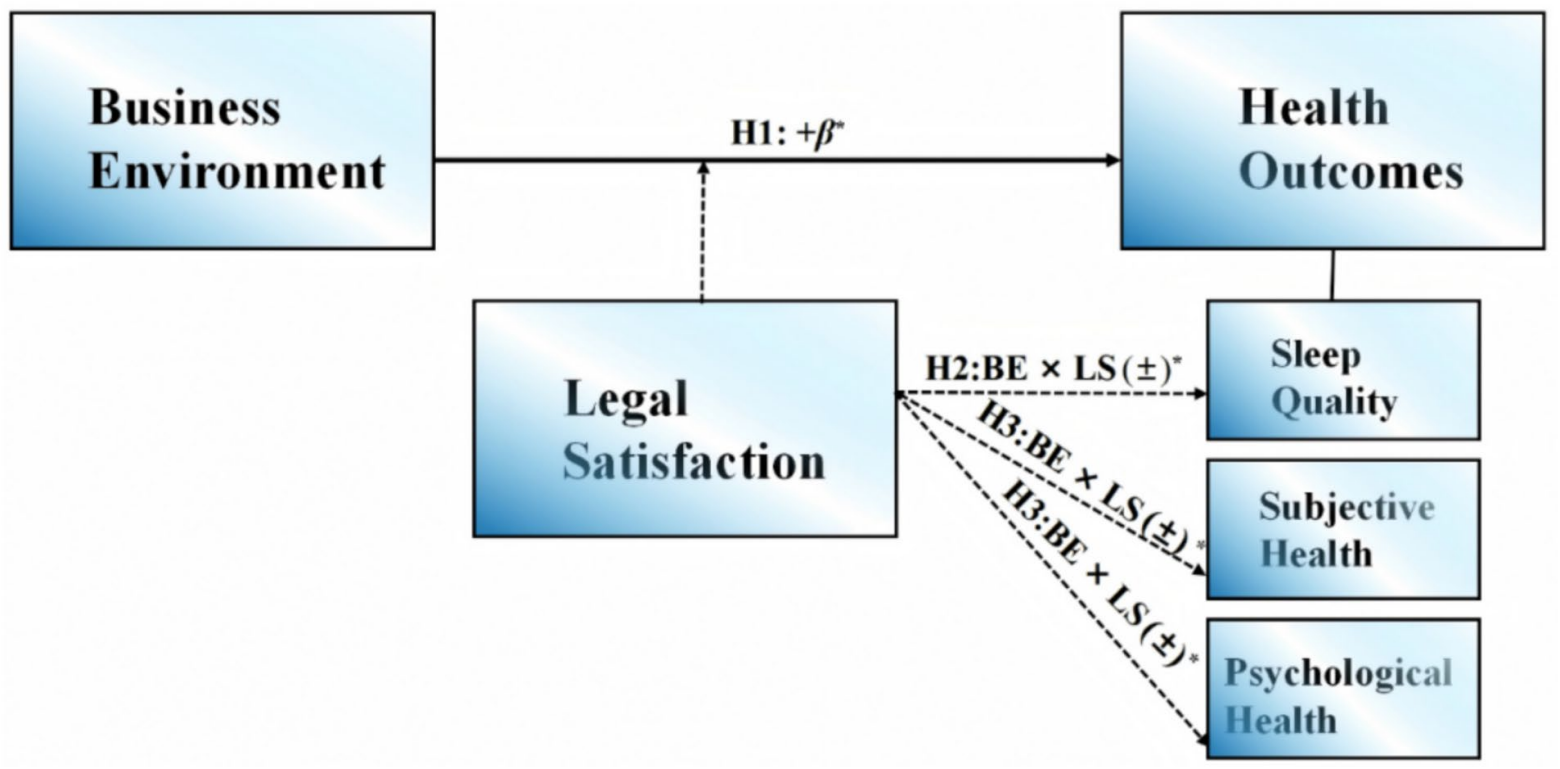
Hypothesis model. BE × LS = Business environment × financial legal satisfaction; *p < 0.05.

The level of the rule of law is a vital factor in assessing the quality of the business environment. Financial professionals’ satisfaction is particularly valuable when assessing innovation dynamics, as their frontline position offers direct insights into regulatory constraints and the effectiveness of policy. For instance, in a study examining the impact of the business environment on corporate investment, researchers analyzed data from 297 company managers in Hangzhou, China, to gather insights from financial professionals in the industry (35). The conservation-of-resources (COR) theory posits that individuals are inherently motivated to acquire and safeguard resources crucial for their survival and well-being. The efficiency of resource utilization within this framework is substantially influenced by an individual’s perception of resource availability and reliability (36). Currently, this theory has been extensively applied to interpret and anticipate work-related stress phenomena. It also serves to dissect the mechanisms underlying the emergence of stress and individual resilience within work environments and organizational cultures (37). COR theory has also been applied in China’s financial industry to explore the personality traits and emotions of financial professionals (38). Nevertheless, investigations concentrating on the health outcomes and well-being of financial professionals remain scarce. Within the COR framework, financial professionals’ legal satisfaction reflects their perception of the availability and reliability of legal resources in a business environment. Those with high financial legal satisfaction show greater trust in the institutional resources of the business environment and are more likely to deploy these related resources actively. When the business environment is optimized, these delighted financial professionals are more likely to quickly take advantage of new policies to reduce their company’s financing costs and ease their overtime stress, which helps regulate their mood and improve their health outcomes. Consequently, exploring the moderating role of financial legal satisfaction will promote the optimization of the business environment and the health outcomes of financial professionals (Figure 1).

### 1.4 Aims

The study aims to (1) assess the health status of financial professionals in China, encompassing sleep quality, subjective health perceptions, and psychological stress levels. (2) examine the association between the business environment and their health outcomes, and (3) the moderating effect of financial legal satisfaction on this association. Consequently, the following hypotheses are formulated:

*H1*: A positive association exists between the business environment and the health outcomes of financial professionals in China.

*H2*: Financial legal satisfaction serves as a moderating role in the relationship between the business environment and the sleep quality of financial professionals.

*H3*: Financial legal satisfaction serves as a moderating role in the relationship between the business environment and the subjective health of financial professionals.

*H4*: Financial legal satisfaction serves as a moderating role in the relationship between the business environment and the psychological stress of financial professionals.

## 2 Materials and methods

### 2.1 Participants and procedures

Data collection took place from November 2024 to January 2025, utilizing online questionnaires with a multi-stage stratified sampling approach among Chinese fintech financial professionals. The samples encompassed various provinces in China, including Heilongjiang, Zhejiang, Jiangsu, Tianjin, and Beijing, as well as other regions. This geographic representation enhances the understanding of diverse developments and professional characteristics within the fintech industry across different regions. The demographic characteristics of the financial professional sample in the study exhibit a high degree of consistency with the data presented in the China Labor Statistics Yearbook, an authoritative annual statistical reference compiled and published by the National Bureau of Statistics of China. Ethical approval for the study was obtained from the Ethics Committee of the Institutional Review Board at Harbin Medical University. In the study, the minimum sample size of 514 was determined according to the calculation method proposed by Zhou et al. (39) Considering the difficulties in achieving a 100% actual response rate and potential issues in questionnaire quality control, a preliminary survey involving 719 participants was conducted, facilitated by the online platform “Questionnaire Star.” Before completing the questionnaire, all participants provided informed consent, as indicated by clicking the “I agree” button at the beginning of the questionnaire. The questionnaire’s inclusion criteria were as follows: (1) professionals in the financial industry, including senior managers, technical R&D staff, marketing personnel, and risk management professionals; (2) voluntary participation in the online survey. To ensure response validity, quality-control questions, such as “How carefully are you answering the questionnaire?,” were incorporated into the questionnaire. Moreover, invalid questionnaires with short response times, numerous missing values, or failure to pass quality-control questions were excluded. The emphasis on fintech professionals was intentional, as this sector is at the forefront of the deep integration of finance and technology. These professionals possess unique expertise that enables them to identify core issues and provide valuable insights for research in specialized subdomains. This study indicates that the risk of common method bias is minimal, as demonstrated by Harman’s single-factor test. Furthermore, the data used in this research comes from multiple sources and is not gathered from a single fintech company’s environment, which helps to further reduce the risk of common method bias (40).

### 2.2 Measurement of health outcomes

Referring to previous studies, psychological stress is used as a measure of physicians’ perceived stress, which is scored on a 5-point Likert scale ranging from 1 to 5 (1 = not at all, 5 = very much), with higher scores representing higher levels of psychological stress (5). Tested across a variety of backgrounds and cultures, Zhang et al. (41) have confirmed that a single-item questionnaire has high validity and sensitivity, and has been used to measure sleep quality and subjective health level in a Chinese nursing context, demonstrating the effectiveness of individual assessments in quantifying subjective health outcomes. Subjective sleep quality can be measured by using an item (42), “How do you assess your sleep tonight?” Scored on a 5-point Likert scale ranging from 1 to 5 (5 = excellent, 4 = very good, 3 = good, 2 = fair, 1 = poor), which provides a visual assessment of sleep quality and may be an effective reflection of the overall health of financial professionals. In addition, our study consulted with Fein and Skinner (43) and estimated overall subjective health status through a widely used single measure (“In general, would you say your health is very good, good, poor, very poor”). A higher score reflects better health. Single-item scales for measuring health outcomes have several advantages, including ease of implementation and straightforward interpretation. However, they also come with several disadvantages. Their simplicity can hinder a deeper understanding of complex constructs, making it essential to consider the context when interpreting the results.

### 2.3 Measurement of business environment

The study developed a business environment evaluation scale based on the “2020 Assessment of Doing Business in Chinese Cities” report. The scientific validity and rationality of the scale construction were strongly supported by the research findings of Du et al. (44). In the context of China’s unique economic environment, the scale has undergone rigorous testing and has been demonstrated to possess sound reliability and validity (45). Moreover, the index system was comprehensively considered, covering seven core dimensions: public service, human resources, market environment, innovation environment, financial services, legal environment, and government affairs environment. Each question within the scale was rated on a 5-point Likert scale, where 1 denoted “completely dissatisfied” and 5 signified “completely satisfied.” Evidently, a higher score indicated a more favorable business environment. In the context of the current study, the scale demonstrated excellent internal consistency reliability, with a Cronbach’s *α* coefficient value of 0.928.

### 2.4 Measurement of financial legal satisfaction

In the study, the financial legal satisfaction of financial professionals related to the business environment in fintech was measured using a single item titled “Are you satisfied with the current laws and regulations related to AI applications in fintech companies?,” adapted from the previously validated Workplace IT Satisfaction Scale, which effectively addresses the study’s focus on their financial legal satisfaction within the financial business environment, demonstrating strong applicability. Responses were recorded on a 5-point Likert scale from 1 (“very dissatisfied”) to 5 (“very satisfied”). The scale demonstrated good reliability and validity within the Chinese cultural context (46), with a Cronbach’s *α* coefficient of 0.765 in this study. As a standalone measure, it does not encompass all elements of financial legal satisfaction for financial professionals and requires further refinement and development to enhance the construct validity and reliability (47).

### 2.5 Data analysis

All statistical analyses were conducted using SPSS version 22.0 (IBM, IBM SPSS Statistics for Windows), with a significance level set at *p* < 0.05 (two-tailed). The Pearson correlation analysis was employed to assess the relationships between continuous variables. Hierarchical multiple regression analysis was employed to examine the relationships between the business environment, financial and legal satisfaction, and health outcomes. Specifically, the study aimed to examine and validate the moderating role of financial legal satisfaction in the relationship between the business environment and health outcomes. Include potential control variables in the first step of the analysis. The second step is to include the variable of business environment. In the third step, the variable of financial legal satisfaction is added, and the last step includes the interaction between financial legal satisfaction and business environment. To visually demonstrate the interaction, a simple slope analysis method is employed. During this analysis, the mean of z values below −1.0 standard deviations and values above +1.0 standard deviations were used as thresholds to divide the high and low levels of continuous moderating variables. In addition, the study rigorously controlled for variables including sex, marital status, age, job tenure, education level, position, and monthly salary income elucidate the health outcomes more precisely. Controlling for sex mitigates the influence of differing social roles associated with sex. Adjusting for marital status reduces the indirect effects of variations in family responsibilities and social support networks. Accounting for age minimizes potential biases arising from intergenerational differences in experience and technological adaptability. Controlling for job tenure is crucial, as prolonged tenure may lead to accumulated burnout, which can influence study outcomes. Moreover, controlling for education level addresses disparities in information access and policy comprehension. Controlling for position can effectively mitigate the potential influences of job hierarchies on resource access, job stress, and policy perception. Finally, controlling for monthly salary income helps reduce the interference of income-level disparities on quality of life, stress perception, and policy dependence.

## 3 Results

### 3.1 Demographic characteristics

The demographic characteristics of the participants and the differences between groups are detailed in the table. Among the financial professionals in this survey, the age range ranged from 15 years old to over 59 years old. Among them, 47.3% were male and 52.7% were female. In terms of marital status, 283 were unmarried, accounting for 55.1%; 201 were married or cohabiting with a partner, accounting for 39.1%; there were 30 divorced or widowed people, accounting for 5.8%. The results showed that sex, marital status, age, and job tenure were significantly associated with health outcomes (*F* = 3.251, *p* < 0.01; *F* = 3.346, *p* < 0.01) (Table 1).

**Table 1:** Sociodemographic differences in participants’ characteristics in health outcomes (*N* = 514).

Variables	*N*	%	Sleep quality	Subjective health	Psychological stress
Sex					
① Male	243	47.3	3.19 ± 1.10	3.39 ± 0.97	2.53 ± 1.05
② Female	271	52.7	3.46 ± 1.00	3.61 ± 0.87	2.47 ± 0.98
*F*/*t*			*t* = 3.022 *p <* 0.01	*t* = 2.689 *p <* 0.01	*t* = −0.610 *p >* 0.05
*LSD*			① *>* ②	① < ②	
Marital status					
① Unmarried	283	55.1	3.36 ± 1.05	3.57 ± 0.93	2.48 ± 0.93
② Married or cohabiting with a partner	201	39.1	3.31 ± 1.06	3.47 ± 0.88	2.55 ± 1.11
③ Divorced or loss of spouse	30	5.8	3.20 ± 1.10	3.03 ± 1.07	2.33 ± 0.99
*F*/*t*			*F* = 0.367 *P >* 0.05	*F* = 4.849 *P <* 0.01	*F* = 0.680 *p >* 0.05
*LSD*				① *>* ③; ② *>* ③	
Age (years)					
① [15 ~ 20)	43	8.4	3.33 ± 1.13	3.53 ± 0.98	2.40 ± 0.88
② [20 ~ 25)	124	24.1	3.56 ± 0.94	3.73 ± 0.82	2.50 ± 0.99
③ [25 ~ 60)	59	11.5	3.47 ± 1.09	3.63 ± 0.87	2.37 ± 1.10
④ [60, ∞)	288	56	3.21 ± 1.07	3.38 ± 0.95	2.54 ± 1.02
*F*/*t*			*F* = 3.596 *P <* 0.05	*F* = 4.712 *P <* 0.01	*F* = 0.602 *P >* 0.05
*LSD*			② *>* ④	② *>* ④	
Job tenure (years)					
① [0 ~ 4)	202	39.3	3.50 ± 1.00	3.63 ± 0.90	2.45 ± 0.97
② [4 ~ 6)	49	9.5	3.33 ± 1.16	3.49 ± 1.04	2.49 ± 0.94
③ [6 ~ 11)	68	13.2	3.37 ± 0.95	3.51 ± 0.92	2.49 ± 1.03
④ [11 ~ 16)	74	14.4	2.95 ± 1.12	3.31 ± 1.01	2.73 ± 1.15
⑤ [16, ∞)	121	23.5	3.28 ± 1.07	3.40 ± 0.85	2.45 ± 1.00
*F*/*t*			*F* = 3.850 *P <* 0.01	*F* = 2.096 *P >* 0.05	*F* = 1.172 *P >* 0.05
*LSD*			① *>* ④; ② *>* ④		
③ *>* ④;④ < ⑤					
Education level					
① College degree or below	81	15.8	3.22 ± 1.27	3.46 ± 1.05	2.33 ± 1.08
② Bachelor’s degree	315	61.3	3.37 ± 0.98	3.53 ± 0.85	2.51 ± 0.94
③ Master’s degree	99	19.3	3.24 ± 1.09	3.45 ± 0.95	2.61 ± 1.07
④ Doctor’s degree	19	3.7	3.58 ± 1.17	3.47 ± 1.39	2.47 ± 1.39
*F*/*t*			*F* = 1.047 *P >* 0.05	*F* = 0.254 *P >* 0.05	*F* = 1.112 *P >* 0.05
Position					
① Senior Management	75	14.6	3.32 ± 0.99	3.45 ± 1.00	2.48 ± 1.04
② R&D Staff	58	11.3	3.17 ± 0.99	3.41 ± 0.82	2.64 ± 1.06
③ Marketing Staff	91	17.7	3.40 ± 0.93	3.51 ± 0.86	2.52 ± 0.97
④ Risk Managers	25	4.9	3.60 ± 1.08	3.28 ± 1.31	2.72 ± 1.02
⑤ Unsure	265	51.6	3.32 ± 1.13	3.55 ± 0.90	2.45 ± 1.00
*F*/*t*			*F* = 0.821 *P >* 0.05	*F* = 0.759 *P >* 0.05	*F* = 0.774 *P >* 0.05
Monthly salary income (RMB)					
① [0 ~ 5,001)	208	40.5	3.32 ± 1.16	3.47 ± 0.95	2.55 ± 1.03
② [5,100 ~ 9,001)	140	27.2	3.24 ± 1.02	3.41 ± 0.93	2.51 ± 1.00
③ [9,001 ~ 15,001)	86	16.7	3.37 ± 0.93	3.58 ± 0.95	2.43 ± 0.94
④ [15,001 ~ 20,001)	45	8.8	3.51 ± 0.87	3.64 ± 0.74	2.40 ± 1.10
⑤ [20,001, ∞)	35	6.8	3.46 ± 1.07	3.69 ± 0.87	2.43 ± 1.04
*F*/*t*			*F* = 0.772 *P >* 0.05	*F* = 1.165 *P >* 0.05	*F* = 0.380 *P >* 0.05

### 3.2 Correlations among continuous variables

**Figure 2:**
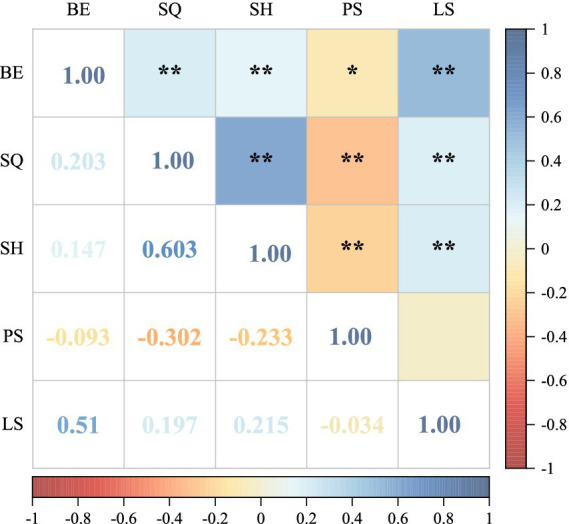
Correlations among continuous variables. BE, business environment; SQ, sleep quality; SH, subjective health; PS, psychological stress; LS, financial legal satisfaction; ^*^p < 0.05; ^**^p < 0.01.

The health outcomes scores of Chinese financial professionals were 3.33 ± 1.06 for sleep quality, 3.50 ± 0.93 for subjective health, and 2.50 ± 1.01 for psychological stress. Figure 2 illustrates the correlations among the business environment, health outcomes, and financial legal satisfaction. The business environment shows a positive correlation with financial legal satisfaction (*r* = 0.510, *p* < 0.01), as well as with sleep quality and subjective health (*r* = 0.203, *p* < 0.01; *r* = 0.147, *p* < 0.01). However, it exhibits a negative correlation with psychological stress (*r* = −0.093, *p* < 0.05).

### 3.3 Hierarchical regression analyses

**Figure 3:**
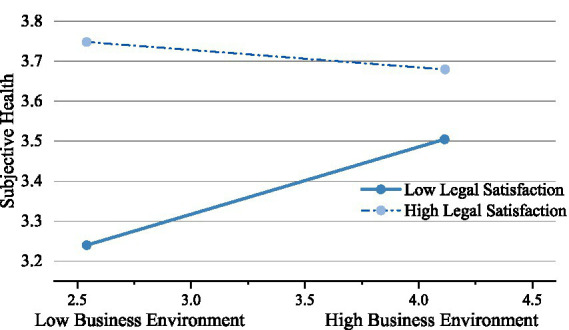
Simple slope plot of the interaction between business environment and financial legal satisfaction on subjective health.

**Figure 4:**
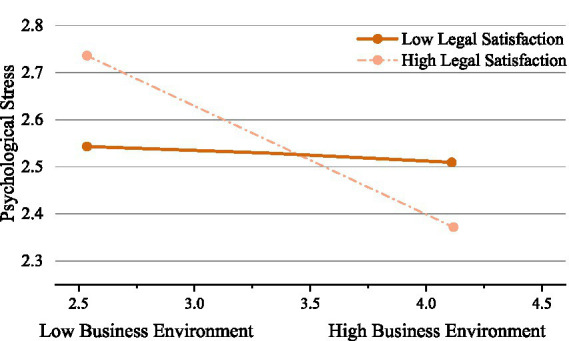
Simple slope plot of the interaction between business environment and financial legal satisfaction on psychological stress.

The results of the analytical regression analysis are presented in Table 2. In the first, fourth, and seventh steps, continuous variables such as sex, marital status, age, job tenure, and education level were included. This is because these variables are highly likely to have an impact on health outcomes, including sleep quality and subjective health. The results of the analysis showed that these control variables had explanatory power for significant differences in sleep quality (*R*^2^ = 0.031, adjusted *R^2^* = 0.021, *p* < 0.01) and significant differences in subjective health (*R^2^* = 0.032, adjusted *R^2^* = 0.022, *p* < 0.01). In the analysis of steps 2, 5, and 8, the business environment is included in the study. The results showed that the business environment was significantly positively correlated with sleep quality (*β* = 0.125, *p* < 0.05) and negatively correlated with psychological stress (*β* = −0.103, *p* < 0.05), which is partially consistent with Hypothesis 1, indicating that the business environment may be beneficial to improving sleep quality and relieving psychological stress. In the third, sixth, and ninth steps, the interactive term of “business environment × financial legal satisfaction” was introduced. This is in view of the fact that financial legal satisfaction may have a moderating effect on the relationship between the business environment and health outcomes. The results showed that the interaction item was significantly negatively correlated with subjective health and psychological stress (*β* = −0.625, *p* < 0.01; *β* = −0.572, *p* < 0.05), which validates Hypothesis 3 and Hypothesis 4; however, Hypothesis 2 remains unsubstantiated. The simple slope analysis further shows that the higher the financial legal satisfaction, the weaker the promotion effect of the business environment on subjective health. The higher the financial legal satisfaction, the stronger the negative impact of the business environment on psychological stress. This suggests that the degree to which the business environment influences subjective health and psychological stress varies according to the individual’s financial legal satisfaction, and this interaction is illustrated in Figures 3, 4.

**Table 2:** Hierarchical multiple regression results of health outcomes in financial professionals (*N* = 514).

Variables	Sleep quality (*β*)			Subjective health (*β*)			Psychological stress (*β*)		
	*M1*	*M2*	*M3*	*M4*	*M5*	*M6*	*M7*	*M8*	*M9*
Control variables									
Sex	−0.099	−0.088	−0.088	−0.101	−0.093	−0.090	0.018	0.013	0.016
Marital status	0.086	0.100	0.099	−0.088	−0.078	−0.068	−0.035	−0.042	−0.032
Age	−0.066	−0.049	−0.049	−0.096	−0.078	−0.079	0.025	0.023	0.022
Job tenure	−0.112	−0.092	−0.092	0.029	0.043	0.037	0.043	0.034	0.029
Education level	0.013	0.017	0.017	−0.021	−0.015	−0.020	0.067	0.069	0.064
Independent variable									
Business environment		0.125^*^	0.099		0.031	0.378^**^		−0.103^*^	0.214
Moderator variable									
Financial legal satisfaction		0.119^*^	0.092		0.178^**^	0.539^**^		0.024	0.354^*^
Interaction									
Business environment × financial legal satisfaction			0.047			−0.625^**^			−0.572^*^
*F*	3.251^**^	5.772^**^	5.046^**^	3.346^**^	5.339^**^	5.678^**^	0.710	1.127	1.741
*R^2^*	0.031^**^	0.074^**^	0.074	0.032^**^	0.069^**^	0.083^**^	0.007	0.015	0.027^*^
*△R^2^*	0.021^**^	0.061^**^	0.059	0.022^**^	0.056^**^	0.068^**^	−0.003	0.002	0.011^*^

^∗^*p* < 0.05; ^∗∗^*p* < 0.01; *β* is the normalized regression coefficient.

## 4 Discussion and conclusion

### 4.1 Current status of health outcomes and their causes among financial professionals

The study investigated the health outcomes of 514 financial professionals and revealed a rather disheartening situation. Specifically, their sleep quality was subpar, subjective health was poor, and psychological stress was overwhelming, highly congruent with the findings in the Chinese Medical Doctor Association and the Chinese Hospital Association “White Paper on the Health of Chinese Financial Professionals” issued by the Chinese Medical Doctor Association. Concurrently, sex, marital status, age, and job tenure were found to influence the health outcomes of financial professionals. Specifically, females had lower sleep quality but higher subjective health than males; both unmarried and married or cohabiting with a partner had better subjective health than those divorced or loss of spouse; individuals aged 20 ~ 25 (inclusive) had better sleep quality and subjective health than those aged 60 and above; those with 11 ~ 16 years (inclusive) of job tenure had lower sleep quality compared to other groups, reflecting the severe physical and mental health challenges faced by financial professionals, while simultaneously shedding light on the potential risks underlying the rapid growth of the industry. Moreover, it indicates that research on the health outcomes of financial professionals must account for the joint effects of gender, age-related physiological changes, such as shifts in hormone levels, the progression of chronic diseases, as well as workplace environmental and occupational risk factors. Currently, the suboptimal health outcomes of these professionals might be intricately linked to complex factors in financial markets, like financial crises and trade wars (48). A thorough analysis enhances understanding of Chinese financial professionals’ health outcomes across temporal and policy contexts, providing critical evidence for intervention strategies. The working environment can affect job satisfaction and mental health (49), and the high-pressure working environment within the financial industry may stand as a pivotal contributor to the health issues among financial professionals. Intense work tasks, exacting performance metrics, and a volatile market environment jointly impose a substantial psychological burden. Financial professionals, being persistently exposed to such conditions, experience the accumulation of burnout, which in turn exacerbates their health conditions (50). Simultaneously, the pronounced work-life imbalance represents a critical and non-negligible factor (51). In China, overtime culture is rife. As per the findings of Liang et al. (52), engaging in overtime work exerts detrimental effects on individual mental well-being. In the financial industry, financial professionals frequently confront recurrent overtime and extended working hours, a circumstance that renders securing sufficient rest and recreational time challenging, thereby may compromising their sleep quality and exerting a profoundly adverse impact on their subjective health. Moreover, the inherent volatility of the financial industry, including market fluctuations, shifts in client demand, and uncertainties in career development, may exacerbate psychological stress. To effectively ameliorate this scenario, financial institutions ought to proactively optimize the internal working environment. Managers of financial institutions should rationally devise and distribute work assignments, institute flexible work arrangements, and place emphasis on fortifying the financial professionals’ health-support infrastructure. This includes establishing a regularized health-assessment mechanism and professional psychological counseling services. Concurrently, financial professionals should adopt self-regulation strategies, including emotion management techniques and structured work-rest schedules, to mitigate stress and safeguard their health outcomes.

### 4.2 Business environment and its positive association with health outcomes among Chinese financial professionals

This study reveals a significant positive correlation between the business environment and the sleep quality and subjective health of financial professionals, as well as a negative correlation with their psychological stress. The study’s findings partially corroborate Hypothesis 1. The amelioration of the business environment may remarkably enhance the health outcomes of financial professionals. This discovery furnishes the academic community with a novel study vantage point to explore the influence of economic and financial factors on the health outcomes of financial professionals. And, it breaks through the limitations of traditional occupational health studies, which mainly focus on lifestyle, occupational stress, and other factors (53). In addition, it also contributes to enriching the theoretical framework of occupational health studies in the financial sector and enhances public health. A good financial business environment means that financial institutions have more stable operations and higher financial performance, which may create more stable jobs and appropriately distribute work tasks, as well as increase employee salaries (54). In this environment, financial professionals do not need to worry excessively about the risk of unemployment. At the same time, they can reduce their dependence on a single project, decrease business uncertainty, and alleviate the pressure of long hours and high-intensity work, thereby effectively alleviating the psychological burden. This stress reduction can help improve sleep quality and subjective health experiences. Furthermore, a superior financial business environment will foster enterprise innovation, stimulating regional financial market growth and enhancing ancillary infrastructure (55). This ecosystem offers financial professionals enhanced leisure options and improved access to healthcare, facilitating stress relief and further optimizing health outcomes. Consequently, it is imperative to leverage the advantages of a high-quality business environment to enhance the health outcomes of financial professionals. The study recommends that government departments need to deepen the reform of the financial sector’s business environment to safeguard the job stability of financial professionals. Simultaneously, financial professionals are expected to capitalize on the high-quality business environment to mitigate their work-related stress.

### 4.3 Financial legal satisfaction negatively moderates the association between business environment and subjective health and psychological stress among Chinese financial professionals

Financial legal satisfaction has been identified as a key potential moderator in the financial industry, which has a significant impact on the association between the business environment and the subjective health and psychological stress of financial professionals. Hypothesis 3 and Hypothesis 4 are also verified, while Hypothesis 2 fails to gain support from the study data. As Figures 3, 4 indicate, as the financial legal satisfaction of financial professionals improves, the promotional effect of the business environment on their subjective health gradually attenuates. Moreover, enhanced financial legal satisfaction also exacerbates the adverse impact of the business environment on the psychological stress of financial professionals. Financial legal satisfaction means that financial professionals operate in a relatively comprehensive legal environment, where financial transactions, business development, and other activities are subject to standardized legal satisfaction. When financial legal satisfaction is high, financial professionals may rely more on legal rules to solve problems in their daily work, and their perceived sensitivity to other positive factors in the business environment is reduced, leading to a decrease in their perceived importance of subjective health. Cognitive dissonance theory (56) suggests that individuals experience psychological discomfort when they hold conflicting beliefs or thoughts. Financial professionals who experience high financial and legal satisfaction may still find themselves dissatisfied with the current business environment. This creates a conflict between their high standards and the reality they observe, leading to psychological stress. Additionally, this high financial legal satisfaction may raise their expectations for an ideal business environment, making them feel disoriented and negatively impacting their overall subjective health. Besides, although high financial legal satisfaction ensures the standardization of financial business, it may also bring more cumbersome compliance requirements and legal risk considerations. Elevated financial legal satisfaction paradoxically intensifies psychological burdens for financial professionals by necessitating greater compliance efforts with complex regulations and subjecting each business decision to stringent legal scrutiny. Positive factors, such as innovation incentives and business development opportunities, that may exist in the original business environment are often overshadowed by the pressure of legal risk prevention under high financial and legal scrutiny, which has a more substantial negative impact on psychological stress. This finding strengthens the current study on the interaction between the business environment and the subjective health and psychological stress of financial professionals in China, advancing theoretical understanding of the mechanisms through which financial legal satisfaction influences health outcomes in this distinctive institutional context.

Considering the negative moderating effect of financial legal satisfaction, government departments should incorporate the balance between legal norms and multiple factors of the business environment into their strategic considerations for policy formulation. In the process of improving the legal system, it is necessary to fully assess its comprehensive impact on the health outcomes of financial professionals, ensuring regulatory frameworks not only satisfy legal standards but also enhance subjective health and reduce psychological stress, thereby fostering sustainable development of China’s financial industry. In an environment of high financial legal satisfaction, financial enterprises should prioritize employee mental health maintenance and stress management, cultivating a supportive work environment to counteract the psychological stress associated with stringent compliance requirements. More significantly, the fintech industry should foster the adaptability and resilience of financial professionals in response to changes in the legal framework. This study recommends that financial professionals establish an active mechanism for continually updating their knowledge. They should regularly participate in industry legal training activities, enhance communication and interaction with peers, and proactively plan their work schedules.

### 4.4 Limitations

Although the study reveals the significant role of financial legal satisfaction in the relationship between the business environment and the subjective health and psychological stress of financial professionals in China, it still has some limitations. Firstly, the cross-sectional design limits the inference of causal relationships and mediating effects (57), and future studies could adopt longitudinal designs or intervention studies to enhance causal interpretability. Secondly, the limited sample coverage does not fully represent the financial industry across all regions of China, and future studies should expand sample diversity to improve the generalizability of the results. Additionally, cultural differences may affect the generalizability of the conclusions, necessitating further validation in cross-cultural contexts. Moreover, the measurement of health outcomes variables is also considered a subjective concept, with certain limitations, and cannot fully capture the objective physiological dimensions of health and other psychosocial dynamics. Lastly, despite implementing strict quality control measures, potential biases in online data collection may still exist, and future studies should optimize data collection methods to enhance reliability. These limitations should be considered when interpreting the results and addressed in future studies.

## 5 Conclusion

Overall, the health outcomes of Chinese financial professionals warrant focused attention, with sex, marital status, age, and job tenure emerging as influential factors. This study’s findings align with China’s strategic initiative to advance a rule-of-law-based business environment, a critical imperative for high-quality economic development. A robust legal-business ecosystem may enhance financial professionals’ financial legal satisfaction, thereby improving their health outcomes and well-being. Such an environment may enable professionals to capitalize on economic opportunities while mitigating occupational stress and safeguarding their physical and mental health. To effectively address this issue, a multifaceted strategy is essential. On the one hand, it is crucial to enhance psychological care and support for financial professionals, reduce working hours reasonably, and optimize the work environment. These measures are expected to improve their health levels, thereby enhancing their health outcomes and increasing their occupational health literacy. The study demonstrates that the business environment is positively associated with the sleep quality, subjective health, and financial legal satisfaction of financial professionals, while it is negatively related to psychological stress. To effectively address this issue, a comprehensive strategy is necessary. First, it is important to enhance psychological care and support for financial professionals, reduce working hours to a reasonable level, and improve the work environment. These measures are expected to boost their overall health, leading to better health outcomes and increased occupational health literacy. The study shows that the business environment positively affects sleep quality, subjective health, and financial legal satisfaction among financial professionals, while it negatively impacts psychological stress. Additionally, financial legal satisfaction exhibits negative moderation in the relationship between the business environment and the subjective health and psychological stress of financial professionals. Crucially, investigating additional determinants of financial professionals’ health outcomes remains imperative for developing targeted interventions, contributing to informing policy optimization to foster healthier work environments, and ultimately enhancing financial professionals’ overall health outcomes. The inherent characteristics of the financial industry should be carefully studied. In recent years, the Chinese government has been strengthening national-level financial regulatory systems (58), which means that financial professionals may face significant regulatory stress. Along with the ongoing improvements in the business environment, these factors create a dual stress from both regulation and the business climate. This combination can impact the health of financial professionals. This study focuses on financial professionals; however, the positive impact of a supportive business environment on health outcomes may also be applicable to practitioners in other fields. Research by Liu et al. (59) indicates that improving the overall business environment can significantly enhance the likelihood of immigrant groups in China’s influx areas participating in medical insurance. This, in turn, may reduce health risks and improve health outcomes. Thus, this study advocates that more experts and scholars delve into the associations between business environment and health outcomes in other domains to enrich the research spectrum of this field. While there are differences in China’s economic system compared to other countries, there are also commonalities in how various nations can improve their business environment. The relevance of this study for financial professionals in other countries still requires further discussion, taking into account factors such as industry characteristics, individual attributes, and cultural context.

## Data Availability

The raw data supporting the conclusions of this article will be made available by the authors, without undue reservation.
